# A rapid and sensitive, multiplex, whole mount RNA fluorescence in situ hybridization and immunohistochemistry protocol

**DOI:** 10.1186/s13007-023-01108-9

**Published:** 2023-11-22

**Authors:** Tian Huang, Bruno Guillotin, Ramin Rahni, Kenneth D. Birnbaum, Doris Wagner

**Affiliations:** 1 Department of Biology, University of Pennsylvania, Philadelphia, PA 19104 USA; 2 Department of Biology, Center for Genomics and Systems Biology, New York University, New York, NY 10003 USA

**Keywords:** RNA-FISH, Hybridization chain reaction, Whole mount, Immunohistochemistry, Fluorescent protein

## Abstract

**Background:**

In the past few years, there has been an explosion in single-cell transcriptomics datasets, yet in vivo confirmation of these datasets is hampered in plants due to lack of robust validation methods. Likewise, modeling of plant development is hampered by paucity of spatial gene expression data. RNA fluorescence in situ hybridization (FISH) enables investigation of gene expression in the context of tissue type. Despite development of FISH methods for plants, easy and reliable whole mount FISH protocols have not yet been reported.

**Results:**

We adapt a 3-day whole mount RNA-FISH method for plant species based on a combination of prior protocols that employs hybridization chain reaction (HCR), which amplifies the probe signal in an antibody-free manner. Our whole mount HCR RNA-FISH method shows expected spatial signals with low background for gene transcripts with known spatial expression patterns in Arabidopsis inflorescences and monocot roots. It allows simultaneous detection of three transcripts in 3D. We also show that HCR RNA-FISH can be combined with endogenous fluorescent protein detection and with our improved immunohistochemistry (IHC) protocol.

**Conclusions:**

The whole mount HCR RNA-FISH and IHC methods allow easy investigation of 3D spatial gene expression patterns in entire plant tissues.

## Background

Profiling spatiotemporal gene expression patterns is critical for studying developmental biology. RNA fluorescence in situ hybridization (FISH) allows detection of spatial gene expression at different developmental stages of an organism. Several types of FISH methods have been established for plants [1–6]. Traditionally, oligonucleotide probes targeting specific transcripts are labeled by epitopes. After hybridizing to the target RNA, the probes are visualized by antibody-based methods [1, 2, 6]. Alternatively, for single molecule FISH (smFISH), short oligonucleotide probes targeting RNA can be conjugated with fluorescent dyes, and the probes are visualized directly after hybridization [3, 4]. Tens of short smFISH probes are designed for one RNA, which allows sensitive detection of a single RNA molecule. Recently, a FISH method with branched DNA (bDNA) amplification was also described in plants [5]. Despite these advances, most existing RNA FISH methods still require sectioning of the tissue, and the samples are processed and visualized on slides, which is laborious and only provides spatial information in two dimensions [2–5]. Rapid and robust methods are needed in plants to corroborate or identify the tissue of origin for single-cell clusters but also generally speed up the workflow of in situ hybridization.

RNA-FISH based on hybridization chain reaction (HCR) has been designed, tested, and optimized in animal species [7–11]. HCR enables antibody-free FISH signal amplification via the self-assembly of small oligonucleotides [12]. A recently improved HCR RNA-FISH method (HCR RNA-FISH v3) was reported to have higher sensitivity and robustness with background suppression in all steps [11]. The ease of multiplexing different HCR probe sets also allows simultaneous detection of multiple RNA species. Furthermore, since no protein is involved in this method, it alleviates possible problems with protein penetration in thick tissues, making whole mount FISH much more feasible.

In this paper, we describe a simple 3-day whole mount RNA-FISH protocol for *Arabidopsis thaliana* (Arabidopsis), *Zea mays* (maize), and *Sorghum bicolor* (Sorghum) using HCR. This protocol allows processing of samples in Eppendorf tubes with limited handling, low hybridization temperature, and probe signal that persists for several days after processing if samples are stored at 4˚C. We show that HCR RNA-FISH can detect known gene expression in whole mount plant tissue—even for genes that are expressed in deep tissue layers—and that we can monitor at least two or three genes simultaneously in maize/sorghum and Arabidopsis, respectively. Additionally, this protocol allows the preservation and detection of expressed fluorescent proteins such as GFP alongside FISH probe signal. Finally, we establish an improved protocol for combined FISH and immunohistochemistry (IHC) that can detect RNA and protein in the same sample. This greatly facilitates the study of mobile proteins or of transcription factors and their targets.

## Results

### Development of a whole mount FISH protocol

**Fig. 1:**
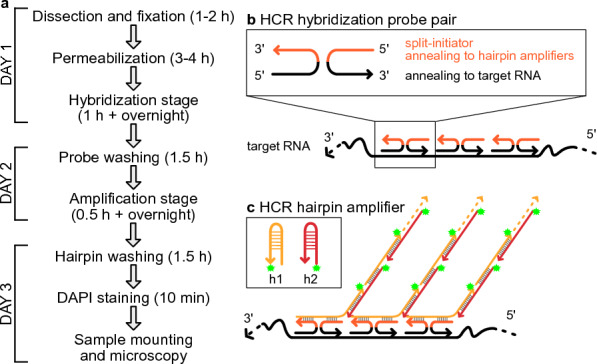
Schematic of plant wholemount HCR RNA-FISH workflow and mechanism. **a** Timeline of 3-day plant wholemount HCR RNA-FISH protocol. **b** In the hybridization stage, HCR hybridization probes anneal to the target RNA, and adjacent probes form an initiator which allows the initiation of the HCR amplification. **c** In the amplification stage, HCR hairpins (h1 and h2) stay self-annealing in the absence of an initiator. When an initiator is present, hairpin h1 and hairpin h2 hybridize to each other and initiate the self-assembly (hybridization chain reaction). Hairpins are tagged by fluorescent dyes (green star), and the self-assembly leads to an amplification of fluorescent signals

We combined and optimized two previously described protocols [1, 11] for whole mount RNA-FISH. To achieve better probe penetration, the cuticle, cell membrane, and cell wall of fixed plant samples are permeabilized through alcohol treatment and cell wall enzyme digestion [1, 13] (Fig. 1a). Next, HCR RNA-FISH is performed on fixed, permeabilized plant samples according to previously described methods in animal species [11] (Fig. 1a). Briefly, probe sets contain multiple hybridization probe pairs that bind different sites on the RNA target. Each probe pairs consist of two small 25 nucleotide single strand DNA probes hybridizing on adjacent sequences of the target mRNA, and each probe contains half of a small DNA initiator sequence. Only when both probes hybridize next to each other can the split-initiators form an intact initiator (Fig. 1b). The initiator triggers the self-assembly of hairpin amplifiers which are tagged by fluorescent dyes, leading to an amplification of fluorescent signal (Fig. 1c). By multiplexing different initiator/amplifier sequences (e.g. B1, B2, B3…) and different fluorescent dyes, simultaneous detection of multiple RNA targets in the same sample can be easily achieved.

**Fig. 2:**
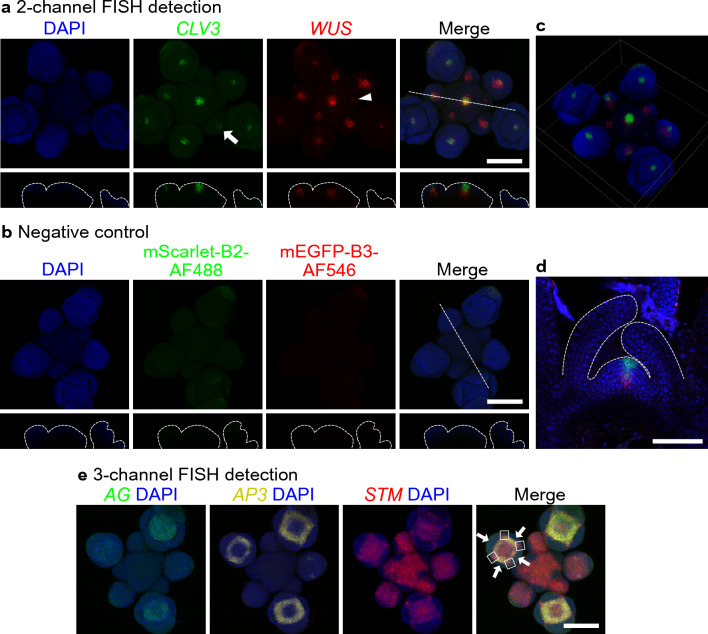
Multiplexed HCR RNA-FISH in wildtype Arabidopsis inflorescence. **a** 2-channel FISH for *CLV3* (green) and *WUS* (red) in inflorescence (top-view with maximum intensity projection). HCR hairpin amplifiers B2-AlexaFlour488 (B2-AF488) and B3-AlexaFlour546 (B3-AF546) were used in the amplification stage. The arrow and arrowhead indicate the earliest flower primordia that express *CLV3* and *WUS* respectively. The orthogonal views across the dash line were shown in the bottom. **b** Test for background. All FISH steps were same as those in Fig. 2a except that mScarletI-B2 and mEGFP-B3 were used in the hybridization step as negative controls. The orthogonal views across the dash line were shown in the bottom. **c** 3D projection of the sample in panel a. **d** FISH for *CLV3* (green) and *WUS* (red) in 11-day-old shoot apical meristem (side-view). **e** 3-channel FISH for *AG* (green), *AP3* (yellow), and *STM* (red) in inflorescence (top-view with maximum intensity projection). HCR hairpin amplifiers B1-AF546, B2-AF488, and B3-AF514 were used in the amplification stage. White arrows indicate the lateral and medial sepal primordia. White squares represent the *STM* expression domain between adjacent sepal primordia. Nuclei were stained by DAPI (blue). Scale bar = 100 µm

To test whether HCR RNA-FISH can detect gene transcripts with known spatial expression pattern, we first chose to examine the expression of the stem cell regulators *CLAVATA3* (*CLV3*) and *WUSCHEL* (*WUS*) in Arabidopsis inflorescences. *CLV3* is expressed in the stem cell niche in the center of the shoot apex, while *WUS* is expressed in the organizing center region below *CLV3* [14, 15]. Wholemount HCR RNA-FISH allowed simultaneous detection of both *WUS* and *CLV3* in a single inflorescence when viewed from above (Fig. 2a). Double labeling showed that *WUS* expression starts to appear in stage 1 flower primordia (Fig. 2a, arrowhead), while *CLV3* expression appears later, in stage 2 flower primordia (Fig. 2a, arrow) (flower stages are determined according to [16]). This agrees with previous observations of *WUS* and *CLV3* temporal expression patterns in flower primordia [14, 15]. Optical longitudinal sections and 3D projection revealed that the *WUS* domain was below the *CLV3* domain, as previously reported [17] (Fig. 2a,c). As a negative control, we employed probes targeting mScarletI and mEGFP and observed a low level of autofluorescence with minimal non-specific binding and amplification (Fig. 2b). Non-specific uniform background was slightly stronger for Alexa Fluor 488 (green) than for Alexa Fluor 546 (red). We also detected *WUS* and *CLV3* expression in young Arabidopsis shoot apical meristem during floral transition using a “half mount” protocol (Fig. 2d). 11-day-old plants were sectioned longitudinally by razor blade through the center of the plant before RNA-FISH. This dissection enables detetction of signal in very young meristems that are buried inside rosette leaves. Similar to the wholemount inflorescence meristem FISH, *CLV3* signal is present above the *WUS* domain, as expected.

Next, we tested the capability of HCR RNA-FISH to detect 3 transcripts simultaneously in the same inflorescence. Using wholemount FISH, *APETALA* 3 (*AP3*), *AGAMOUS* (*AG*), and *SHOOT MERISTEMLESS* (*STM*) were simultaneously probed and detected with previously reported spatial expression pattern [18–22] (Fig. 2e). *AP3* expression occurred one primordium prior to that of *AG*. *AG* expression partially overlapped with that of *AP3*, as expected since *AP3* and *AG* together specify stamen identity [23]. *STM* expression in the meristem was excluded from incipient and very young (< stage 1) flower primordia, as expected [21, 22]. *STM* was expressed in older flower primordia, but was excluded from the lateral and medial sepal primordia (Fig. 2e, white arrows), as previously observed [22]. From stage 3 onward, *STM* expression in flower primordia was entirely contained within the regions demarcated by *AP3* and *AG*, with the exception of the boundaries between the sepal primordia (white squares, see also Long 2000 [22]).

In conclusion, the results of HCR RNA-FISH show expected spatiotemporal gene expression pattern with low background in Arabidopsis inflorescences. Also, HCR RNA-FISH allows simultaneous detection of 2 or 3 different gene transcripts in the same sample.

### Simultaneous FISH and detection of endogenous fluorescent reporters

**Fig. 3:**
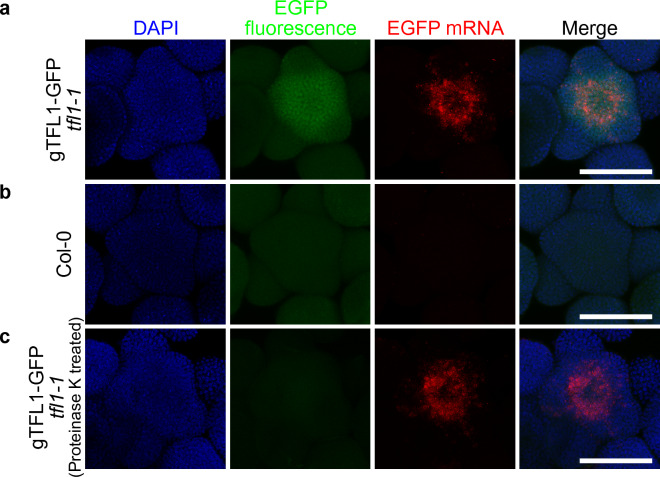
HCR RNA-FISH in the inflorescence of Arabidopsis transgenic reporter. EGFP fluorescence (green) and RNA-FISH for *EGFP* transcripts (red) were detected in gTFL1-GFP *tfl1-1* (**a**), Col-0 wild type (**b**) (top-view with maximum intensity projection). The detection in Col-0 wild type served as a negative control. **c** EGFP fluorescence (green) and FISH for EGFP transcripts (red) in gTFL1-GFP *tfl1-1* with proteinase K treatment (top-view with maximum intensity projection). Nuclei were stained by DAPI (blue). Scale bar = 100 µm

To test whether HCR RNA-FISH can be used together with fluorescent reporters, we monitored transgene expression in null mutants for the *TERMINAL FLOWER 1* (*TFL1*) gene rescued by a translational protein fusion to the EGFP fluorescent protein (gTFL1-GFP *tfl1-1* [24]) as it is known that TFL1 protein moves beyond its site of transcription [25, 26]. For FISH we used probes targeting *EGFP* mRNA and—to avoid possible bleed-through between channels—we chose probes with a fluorescent dye (Alexa Fluor 546) whose excitation and emission spectra do not overlap with EGFP fluorescence. *EGFP* mRNA was detected in the center of the meristem in gTFL1-GFP *tfl1-1*, while little signal was observed in wild type plants using the same probes, as expected (Fig. 3b). This absence of off target binding highlights the high specificity of the signal detected using HCR FISH. The pattern of *EGFP* transcript in gTFL1-GFP *tfl1-1* resembled that of the endogenous *TFL1* transcript in the wild type, which is restricted to the center of the inflorescence meristem [27]. Interestingly, despite methanol and ethanol dehydration in the FISH protocol, we were still able to detect EGFP fluorescence, albeit apparently much lower intensity than in tissues not subjected to FISH. Nevertheless, gTFL1-GFP fluorescence was detected in the previously reported protein accumulation domain [24] simultaneously alongside *EGFP* mRNA. Simultaneous detection of protein fluorescence and RNA allows direct comparison of the protein and RNA expression domains of mobile proteins—like TFL1—which are common in plants [25, 26, 28]. It also allows simultaneous visualization of other reporters (for hormones, subcellular compartments, etc.) alongside transcripts. Conversely, to avoid potential overlap between the excitation and emission spectra of the fluorescent protein and the FISH probe, the fluorescent protein can be easily removed by treating the tissue with proteinase K prior to FISH (Fig. 3).

### Combined FISH and IHC

**Fig. 4:**
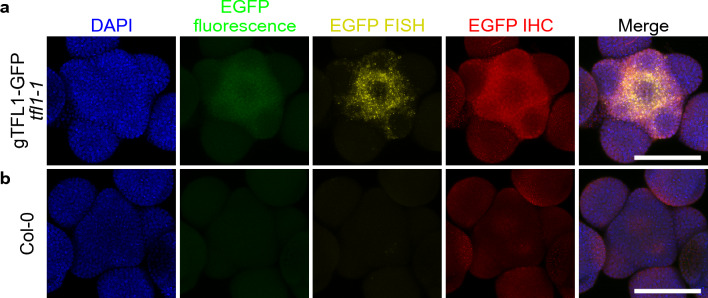
Combined FISH and IHC in Arabidopsis inflorescence. EGFP fluorescence (green), RNA-FISH for *EGFP* transcripts (yellow), and anti-GFP IHC were detected in gTFL1-GFP *tfl1-1* (**a**), Col-0 wild type (**b**) (top-view with maximum intensity projection). The detection in Col-0 wild type served as a negative control. Nuclei were stained by DAPI (blue). Scale bar = 100 µm

Whole mount immunohistochemistry (IHC) methods have been described for Arabidopsis inflorescence [29, 30]. IHC allows detection of proteins whenever an antibody against the target protein or the epitope tag is available. A combined HCR RNA-FISH and IHC method has been recently established in animal species to visualize RNA and protein simultaneously [31]. To test whether HCR RNA-FISH can be combined with IHC in plants, we attempted to simultaneously detect *EGFP* mRNA and protein in gTFL1-GFP *tfl1-1* using *EGFP* RNA-FISH and anti-GFP IHC (Fig. 4). A standard HCR RNA-FISH was performed to detect *EGFP* mRNA. Next, extra cell wall digestion and post-fixation were applied to the samples to achieve higher permeability for antibodies in the IHC. Blocking, primary antibody incubation, and secondary antibody incubation were then performed similar to published IHC methods. We chose Alexa Fluor 514 for RNA-FISH and Alexa Fluor 546 for secondary antibody in IHC so that the spectra of both fluorescent dyes do not overlap with each other and do not overlap with EGFP fluorescence. As shown in Fig. 4, EGFP protein was detected by anti-GFP IHC (red) in the meristem of gTFL1-GFP *tfl1-1*, and only a weak background was detected in the Col-0 wild type negative control. We noticed EGFP fluorescence (green) was also partially maintained, and the pattern of IHC signal (red) resembled that of the EGFP fluorescence (green). At the same time, *EGFP* mRNA was detected in gTFL1-GFP *tfl1-1* by HCR RNA-FISH (Fig. 4, yellow). In Col-0 wild type negative control, EGFP fluorescence (green) and RNA (yellow) were not detectable, as expected. These results suggested that multiplexing IHC and HCR RNA-FISH had good preservation of FISH signal and showed the expected IHC signal pattern. Although we chose EGFP as the target for IHC detection, all kinds of epitope tags should be compatible with this method as long as the chosen primary antibody shows little non-specific binding (low background). When antibodies against endogenous proteins are available, it is possible to simultaneously probe the endogenous proteins with other mRNA targets in non-transgenic plants. In summary, we successfully combined the HCR RNA-FISH protocol with immunohistochemistry which allows simultaneous detection of RNA and protein in whole mount tissue.

### “Half Mount” FISH in monocot roots

Traditional in situ hybridization in monocot roots [32] has involved sectioning, which is challenging and time consuming, and whole mount protocols are not currently available for roots. Here, we adapted the HCR protocol on 3D root tissue in *Zea mays* (maize) and *Sorghum bicolor* (Sorghum). Maize and Sorghum seedlings were grown on germination paper, and a few drops of fixative was applied directly to root tips with a pipette, just prior to hand-sectioning along the longitudinal or transverse axis using a microscalpel. Then the tip is excised and directly transferred into fixative solution. While the fixative solution as well as the processing steps prior to HCR differed for monocot samples (see Methods), the HCR RNA-FISH steps closely follow that described for Arabidopsis above. The resulting “half mount” protocol allowed for clear visualization of HCR probe signal and owing to maize and Sorghum roots’ cell wall autofluorescence, no DAPI counterstain was necessary.

Probes can be visualized with several Alexa fluorophores (Alexa Fluor 488, 514, 546, 594 and 647). We first tested tissue autofluorescence at the excitation wavelengths for each of the fluorophores, determining that excitation of Alexa Fluor 647 and 488 generated the lowest background fluorescence in maize and sorghum roots. Monocots cell walls were visualized using the strong autofluorescence of the tissue under 405 nm laser excitation.

**Fig. 5:**
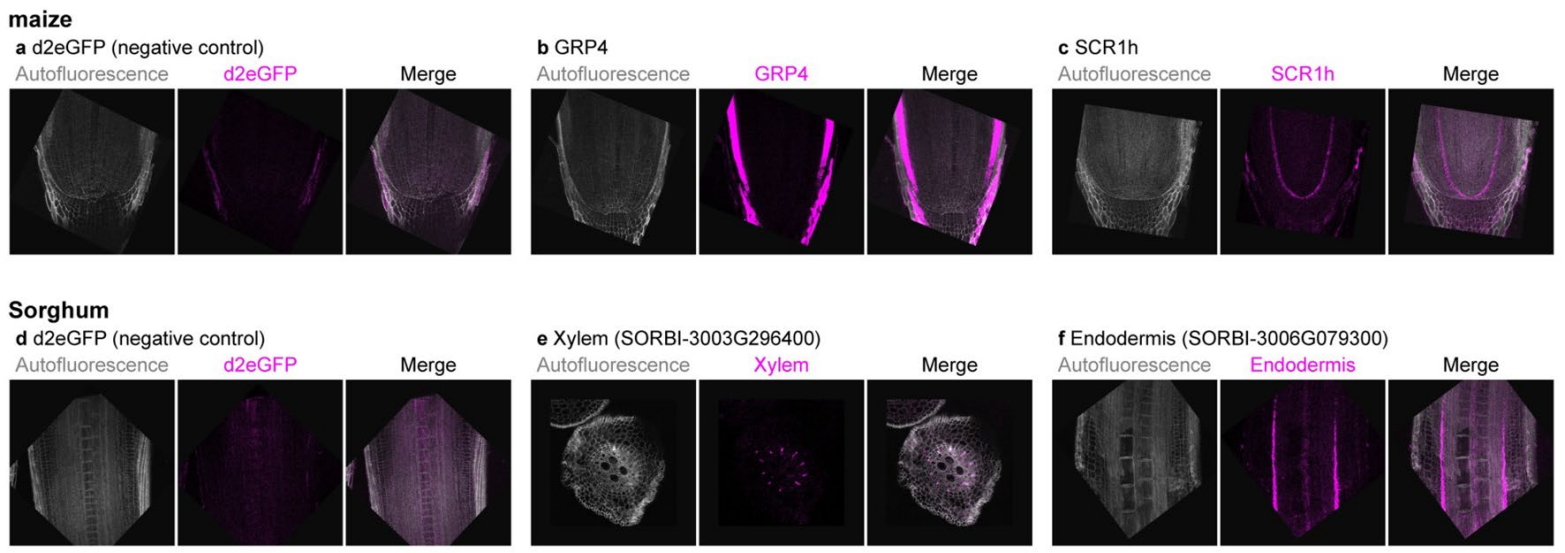
HCR RNA-FISH in monocot roots. HCR RNA-FISH signal in magenta, and cell wall autofluorescence in gray, in either maize (**a**–**c**) or sorghum (**d**–**f**) roots. a,d FISH background fluorescence was assessed using RNA-FISH for *eGFP* transcripts, not expressed in the roots. b,c longitudinal hand sectioning of maize root tips, revealing the signal for FISH against GRP4 or SCR1h maize genes, specifically expressed in lateral root cap/epidermis (**b**) or endodermis (**c**). (**e**) transversal and longitudinal (**f**) hand section of sorghum root, RNA-FISH for SORBI-3003G296400 specifically expressed in xylem (**e**) or for SORBI-3003G079300 expressed in endodermis (**f**)

To test if HCR RNA-FISH can detect maize transcripts with known spatial expression patterns, we examined ZmGRP4 which has reported expression in the lateral root cap and epidermis [32]. We reliably detected strong signal from these tissues, whereas negative control probes targeting GFP showed little to no background signal (Fig. 5a, b). In addition, a probe against SCR1h had an expression pattern matching published in situ hybridization data (Fig. 5c) [33].

Deploying the same protocol in Sorghum, we were able to visualize Xylem and Endodermis markers predicted from single cell analyses [34], with negative control probes showing little to no background signal (Fig. 5d, e).

Thus, this protocol allows for a quick and easy imaging of RNA probes in maize and Sorghum root tissue without the need for microtome sectioning. The protocol greatly speeds up in situ hybridization experiments and allows for a sensitive readout of spatial gene expression, even in normally optically inaccessible thick tissues.

## Discussion

With the rapid development of single-cell transcriptomics techniques in plants [35, 36], our knowledge of tissue specific expression of know regulators has increased dramatically, and an increasing number of unknown genes have been identified that are expressed in specific cell types. However, single-cell transcriptomic data lose the spatial cell to cell contact information due to the dissociation of cells, so orthogonal approaches are needed to validate the spatial expression pattern and derive biological meaning for the genes identified in single-cell transcriptomic data. This increases the need for testing gene expression patterns by in situ hybridization-based methods. Thus far, most approaches used rely on thin sections [2–5, 37], which preclude 3D visualization of transcript accumulation and greatly add to the time it takes to perform localization assays. Here we provide a robust, versatile, and facile wholemount RNA FISH method based on hybridization chain reaction which provides a fast and reliable readout for investigating gene expression pattern in complex plant tissues. The method is rapid and allows for a highly sensitive and specific readout of transcript localization. In addition, unlike existing wholemount in situ hybridization methods [1, 6, 38], it allows simultaneous detection of multiple transcripts. The multiplexing of different gene transcripts is straightforward due to the nature of the HCR technology. We successfully detected *AP3*, *AG*, and *STM* transcripts in the same inflorescence sample, and simultaneous detection for 4 targets is possible [11]. Moreover, the HCR RNA-FISH method is quantitative which has been demonstrated previously for wholemount animal tissues [11]. Although other high-throughput spatial transcriptomics techniques used in plants (e.g. in situ sequencing, or MERFISH) can quantitatively and spatially detect much more transcripts at the same time, they are so far not compatible with wholemount samples [39–42]. We demonstrated that HCR RNA-FISH detected gene expression with precise spatial pattern and low background in wholemount Arabidopsis inflorescences and monocot roots. Also, the partial persistence of fluorescent protein signal and combined FISH and IHC allow co-detection of the transcript and protein of a gene as we shown for mobile protein TFL1.

## Conclusions

The 3-day HCR wholemount RNA-FISH method we described here will facilitate the investigation of spatial gene expression pattern in plant species in 3D.

## Methods

### Hybridization probes and amplification hairpins

Hybridization probes with split-initiators (e.g. B1, B2, B3) targeting specific transcripts were designed and manufactured by Molecular Instruments based on the transcript sequences we provided [11]. According to our experience, most probes worked without further optimization. The synthesized HCR probes recapitulate published expression patterns and very low background is observed in the absence of the transcript (mEGFP, mScarletI probes in the wild type) (Fig. 2). Amplification hairpins with certain amplifier sequences (e.g. B1, B2, B3) and fluorescent dyes were purchased from Molecular Instruments. Probes can be designed to be compatible with Alexa Fluor 488, 514, 546, 594 or 647. All hybridization probes and amplification hairpins used in this study were listed in Table 1. mEGFP probes were used to detect EGFP transcripts since mEGFP and EGFP only have 1 nt difference in sequence.

**Table 1:** Hybridization probes

Transcripts	Initiator/amplifier-fluorescent dye	Lot number
*WUS* (*AT2G17950*)	B3 Alexa Fluor 546	PRM274
*CLV3* (*AT2G27250*)	B2 Alexa Fluor 488	PRM273
*AG* (*AT4G18960*)	B2 Alexa Fluor 488	PRM277
*AP3* (*AT3G54340*)	B3 Alexa Fluor 514	PRM276
*STM* (*AT1G62360*)	B1 Alexa Fluor 546	PRM275
mEGFP	B3 Alexa Fluor 546 or 514	PRK551
mScarlet-I	B2 Alexa Fluor 488	PRN494
d2eGFP	B1 Alexa Fluor 647	PRA221
ZmGRP4 (Zm00001d004728/GRMZM2G025205)	B1 Alexa Fluor 647	PRK923
ZmSCR1h(Zm00001d052380)	B1 Alexa Fluor 647	PRK918
SORBI-3003G296400 (xylem)	B1 Alexa Fluor 647	PRK925
SORBI-3006G079300 (endodermis)	B1 Alexa Fluor 647	PRM013

### Reagents

Fixative solutions: (Arabidospis) 4% paraformaldehyde (PFA) (SIGMA, P6148) in phosphate buffered saline (PBS); (Monocot) FAA: 4% formaldehyde, 5% glacial acetic acid, 50% ethanol in RNAse free water.

50% Histo-Clear II / 50% ethanol: Histo-Clear II (Electron Microscopy Sciences, 64111-01) and ethanol were mixed at 1:1 ratio.

DPBST: fresh-made DPBS with 0.1% Tween-20 (Bio-Rad, 170-6531). DPBS was prepared from 10 × DPBS without calcium and magnesium (gibco, 14200-075).

5 × SSCT: fresh-made 5 × SSC buffer with 0.1% Tween-20 (Bio-Rad, 170-6531). 5 × SSC buffer was prepared from 20 × SSC buffer (CORNING, 46-020-CM).

Cell wall digestion enzyme mix A: The cell wall enzyme mix A formula was adapted from previous publication [1]. First, to prepare 6 × cell wall digestion enzyme mix A stock, 50 mg Macerozyme R-10 (RPI, M22010), 50 mg Cellulose RS (RPI, C32400), 25 mg Pectolyase (SIGMA, P3026), and 1 mL Pectinase (SIGMA, P4716) were dissolved in 10 mL pure water. The stock was filtered through 0.22 µm syringe filter and stored at -20 °C freezer. To prepare 1 × cell wall digestion enzyme mix A, 6 × stock was diluted in DPBST.

Cell wall digestion enzyme mix B (for IHC): The cell wall enzyme mix B formula was adapted from previous IHC protocols [29, 30]. 2 × cell wall digestion enzyme mix B stock (0.4% Driselase (SIGMA, D8037) and 0.3% Macerozyme R-10 (RPI, M22010) in PBS) was prepared and stored at − 20 °C freezer. 2 × stock was diluted in DPBST to prepare 1 × cell wall digestion enzyme mix B.

Proteinase solution: 4 µL proteinase K (NEB, P8107S) was added into 1 mL 0.1 M Tris–HCl 0.05 M EDTA (pH 8.0) to prepare the proteinase solution. The buffer (0.1 M Tris–HCl 0.05 M EDTA pH 8.0) was prepared fresh using 1 M Tris–HCl (pH 8.0) (Invitrogen, 15568-025) and 0.5 M EDTA (pH 8.0) (Invitrogen, 15575-020).

DAPI staining solution: 1 µg/mL DAPI (SIGMA, D9542) in DPBS.

Blocking buffer: 2% bovine serum albumin (SIGMA, A3059) in DPBST.

Primary antibody solution: diluted primary antibody in blocking buffer. For EGFP IHC, rabbit anti-GFP antibody (abcam, ab290) was diluted at 1:2000 in blocking buffer.

Secondary antibody solution: diluted secondary antibody in blocking buffer. For EGFP IHC, Alexa Fluor 546 goat anti-Rabbit IgG antibody (Invitrogen, A11035) was diluted at 1:200 in blocking buffer.

### Plant growth conditions

Arabidopsis plants were grown in soil at 22 °C under long-day photoperiod (16 h light/8 h dark with light intensity of 120 μmol/m^2^s). gTFL1-GFP *tfl1-1* was described in previous publications [26]. All Arabidopsis plants in this study are Columbia-0 ecotype.

Maize and Sorghum seedlings were surface sterilized for 20 min with 6% active chloride, washed with sterile water then grown on germination paper (Anchor Paper&Cie., 38# regular) in tap water (28 °C/24 °C, 16 h light/8 h dark with light intensity of 420 μmol/m^2^s) for 7 days.

### Sample dissection and fixation

Arabidopsis: Shoot apices were collected shortly after bolting. All flowers covering the inflorescence meristem were dissected and removed by forceps or a needle. It is critical to expose the tissue of interest as much as possible, otherwise confocal laser scanning microscope will not be able to capture signals from tissue of interest. The fixative solution (4% PFA in PBS) was prepared in 1.5 mL centrifuge tube in a fume hood. Samples can be fixed with FAA as well, but FAA might reduce the fluorescent protein signal when co-detecting RNA and fluorescent protein signals. Samples were collected in the fixative immediately after dissection. After vacuum infiltration, samples were fixed for another 30 min at room temperature in the fixative. Fixative was removed by washing once in DPBS for 10 min.

Monocots: The fixative solution (FAA) was prepared in 5 mL tube in a fume hood. Just prior to fixation a small volume of fixative FAA was applied directly to the roots using a pipette. Using a microscalpel, roughly 1 cm longitudinal cuts were made in the root tissue, before excising 1.5–2 cm of the root and transferring to fixative solution. Transverse sections, by contrast, were performed just prior to imaging. In fume hood, apply a gentle vacuum until roots float up. Release vacuum, agitate tube, and apply vacuum again. Repeat several times until roots no longer float up (may take up to an hour). Make sure samples are in FAA at room temperature for at least 1 h. Samples can also be stored in FAA overnight at 4 °C.

Tips: Although HCR RNA-FISH allows detection in deeper regions of tissue, the imaging depth is still restricted to about 100 µm due to light absorption and scattering [43]. Thus, sample dissection is critical for exposing the tissue of interest, although we note that all dissections in both Arabidopsis and monocot species shown here were performed by hand under a stereoscope scope. The dissection procedure for FISH is similar to the dissection required for the conventional fluorescence microscopy. To image deeper tissue without dissection, it is possible to further clear the tissue or combine HCR RNA-FISH with multiphoton microscopy [44–47]. Dissection is not required for tissues that are not obscured by other plant structures or small tissues.

### Sample permeabilization Arabidopsis

Cuticle and cell membrane can be permeabilized by series of methanol and ethanol incubation [13]. Steps for sample permeabilization was modified from previous FISH protocols [1].

1. Fixed and washed samples were directly dehydrated in methanol twice for 10 min.
  *PAUSE POINT: samples can be stored in methanol at − 20°C for days before continuing.*
2. Then samples were incubated in ethanol twice for 10 min.
3. Samples were cleared and permeabilized in 50% Histo-Clear II/50% ethanol for 30 min.
4. Samples were then washed in ethanol twice for 10 min and in methanol three times for 5 min.
5. Samples were rehydrated by sequential washing in 75%, 50%, 25%, 0% methanol in DPBST (5 min each).
6. Partial cell wall digestion was then performed by previously described enzyme mix [1]. Samples were incubated for 3 min at room temperature in 1 × cell wall digestion enzyme mix A in DPBST. The incubation time for cell wall digestion needs to be adjusted for different tissues, and excessive cell wall digestion often leads to damaged sample structures and diminished FISH signals according to our experience. (Cell wall digestion can be skipped if it shows good FISH signal without this step.)
7. After cell wall digestion, enzymes were removed by three 2-min washes in DPBST.
8. Then samples were fixed in 4% PFA in PBS for 30 min at room temperature.
9. The fixative was removed by washing the samples in DPBST twice for 5 min.

### Sample permeabilization monocots

1. Dehydrate the samples in a series of washes at room temperature with a tube revolver: 70% ethanol for 15 min, 90% ethanol for 15 min, 100% ethanol twice for 15 min each, 100% methanol twice for 15 min each. Leave samples in methanol at − 20 °C overnight.
  *PAUSE POINT: samples can be stored in methanol at -20 °C for several weeks before continuing.*
2. Incubate twice for 30 min in a solution of 100% Histo-Clear II at room temperature. Each time, apply vacuum for the first 10 min then transfer to a tube revolver for the last 20 min. Rehydrate the samples through a series of washes at room temperature with a tube revolver: 50% Histo-Clear II / 50% ethanol for 15 min, 100% ethanol for 15 min, 50% ethanol / 50% DPBST for 15 min—roots will float up then settle after a few minutes, then 100% DPBST twice for 15 min—roots will float up then settle after a few minutes.
3. Incubate with 4% formaldehyde in DPBST at room temperature under gentle vacuum in fume hood for 10 min. Fix the meristem for 20 min in 4% formaldehyde in DPBST at room temperature on a tube revolver.
4. Wash twice for 15 min each in DPBST at room temperature with a tube revolver.
5. Aliquot roots into 2 mL Eppendorf tubes. Use between 5–10 roots per tube/probe.

### Proteinase K treatment

Proteinase K treatment can be performed after permeabilization if fluorescence from fluorescent proteins needs to be removed. Samples were treated with proteinase solution at 37 °C for 15 min and then washed by DPBST 3 times for 2 min. The digested samples were fixed in 4% PFA in PBS for 30 min and washed in DPBST twice for 5 min.

### HCR RNA-FISH Hybridization

HCR RNA-FISH was performed according to the previous publication [11] and online protocols provided by Molecular Instruments (https://www.molecularinstruments.com/hcr-rnafish-protocols).

Probe solution was prepared by adding 0.4 µL (1 μM stock) of each hybridization probe set into 100 µL pre-heated HCR Probe Hybridization Buffer (Molecular Instruments) at 37 °C.

*Arabidopsis*: Remove DPBST and replace with 200 µL pre-heated HCR Probe Hybridization Buffer (no probe). Samples were incubated in HCR Probe Hybridization Buffer for 30 min at 37 °C. Remove the Hybridization Buffer and add 100 µL probe solution. Samples were then incubated in probe solution overnight (~ 20 h) at 37 °C.

*Monocots*: Remove DPBST and replace with 500 µL of HCR Probe Hybridization Buffer (no probe). Apply gentle vacuum in fume hood for 10 min, then pre-hybridize by incubating for 1 h at 37˚C in a thermomixer with agitation at 1000 rpm. Remove Hybridization Buffer and add the probe solution. Hybridize by incubating overnight (~ 20 h) at 37˚C in a thermomixer with agitation at 1000 rpm.

*PAUSE POINT: before replacing the HCR Probe Hybridization Buffer with the probe solution, samples can be stored in HCR Probe Hybridization Buffer at –20˚C for several weeks before continuing.*

### HCR RNA-FISH amplification

After the HCR RNA-FISH hybridization, samples were washed with pre-heated HCR Probe Wash Buffer (Molecular Instruments) at 37 °C four times for 15 min. Then samples were washed by 5 × SSCT twice for 5 min. For Arabidopsis, samples were then pre-amplified with 200 µL HCR Amplification Buffer (Molecular Instruments) at room temperature for 10 min. For monocots, 5 × SSCT was replaced by 500 µL HCR Amplification Buffer, and then gentle vacuum was applied in fume hood for 10 min. Monocots samples were pre-amplified in tube rotator at room temperature for 50 min.

While samples wash and pre-amplify, the hairpin solution is prepared. For Arabidopsis, 50 µL hairpin solution is prepared for each sample. For monocots, 250 µL hairpin solution is prepared for each sample. For each 50 µL hairpin solution, 3 pmol hairpin h1 and 3 pmol hairpin h2 (i.e. 1 µL of the 3 µM stocks) were separately incubated at 95 °C for 90 s and cooled to room temperature in the dark for 30 min. The hairpin solution is prepared by combining snap-cooled h1 and h2 hairpins in 50 µL of HCR Amplification Buffer at room temperature.

After the pre-amplification, the HCR Amplification Buffer was removed, and samples were incubated in hairpin solution overnight (~ 20 h) in the dark at room temperature.

Excessive hairpins were removed by washing in (1) 5 × SSCT twice for 5 min, (2) 5 × SSCT twice for 30 min, (3) 5 × SSCT once for 5 min.

Samples can be kept in 5 × SSCT for over one or two weeks at 4 °C without any signal losses (depending on the probe brightness).

### DAPI staining for Arabidopsis inflorescences

Samples were washed in DPBST once for 10 min before staining. To stain the nuclei, samples were incubated in DAPI staining solution for 10 min and washed by DPBS. Samples can be stored in DPBS at 4 °C in the dark for at least several days before microscopy.

### Sample mounting and microscopy

*Arabidopsis inflorescences*: Samples were mounted on 2% agarose gel in 60 mm petri dishes. The stem underneath the apex was gently inserted in agarose gel under a stereomicroscope using fine forceps. Mounted samples were submerged in water and imaged by an upright Leica Stellaris 5 White Light Laser confocal microscope equipped with a water immersion objective (HC PL APO 40 × /1.10 W CORR CS2) without a coverslip. A z-stack was captured from the top layer of the shoot apex to the deeper tissue. The x–y resolution for each slice is 1024 × 1024. Details of confocal microscope settings for each figure were shown in Table 2. Maximum intensity projection, rotation, and orthogonal view were performed by FIJI [48]. 3D projection in Fig. 2c was generated by Leica LAS X 3D Visualisation software.

**Table 2:** Confocal microscopy image acquisition settings

	Channel	Laser	Detection	Z-stack	Notes
Figure 2a–d	Blue	405 nm (diode laser)	425–480 nm	50 slices with step size of 2 µm (Fig. 2a–c); none (Fig. 2d)	
	Green	499 nm	505–550 nm		
	Red	557 nm	565–610 nm		
Figures 2e and 4	Blue	405 nm (diode laser)	425–480 nm	20 slices with step size of 4 µm (Fig. 2e); 25 slices with step size of 2 µm (Fig. 4)	The brightness and contrast were adjusted by FIJI
	Green	488 nm	495–525 nm		
	Yellow	520 nm	535–565 nm		
	Red	557 nm	565–610 nm		
Figure 3	Blue	405 nm (diode laser)	425–480 nm	30 slices with step size of 2 µm	The brightness and contrast of green channel were adjusted by FIJI in the same way across panels
	Green	488 nm	495–535 nm		
	Red	557 nm	565–610 nm		
Figure 5	Grey	405 nm	419–800 nm	none	Monocot cell wall autofluorescence
	Magenta	635 nm	649–791 nm	none	The brightness and contrast were adjusted by FIJI

*Monocots*: Transfer samples onto a glass slide (in 5 × SSCT) and using a 15˚ microscalpel cut and arrange them so that the cut face of the roots is facing upwards before being covered with coverslip. Samples were imaged on ﻿Leica SPE inverted confocal microscope, using an ﻿air objective (20 × /0.7) with 2.5 × zoom within the LAS AF software (find details in Table 2).

Tips: After completing the HCR RNA-FISH procedures, small samples tend to be very fragile. The handling of the sample during the mounting step needs to avoid damage to the tissue of interest. For inflorescence samples, we also note that sample movement during image acquisition could happen if samples are not well inserted into agarose gel plates.

### Combined FISH and IHC

In combined FISH and IHC, HCR RNA-FISH was performed as indicated above, and IHC was immediately followed after HCR RNA-FISH. We adapted the IHC protocol from previous published IHC methods [29, 30].

1. After the final 5 × SSCT wash step in the HCR RNA-FISH protocol, samples were washed for 10 min in DPBST.
2. Then the samples were fixed in 4% PFA in PBS for 15 min and washed by DPBST twice for 2 min.
3. To achieve better permeability for antibodies, the cell wall of the samples was further digested with cell wall digestion enzyme mix B for 5 min at room temperature.
4. Digested samples were fixed in 4% PFA in PBS for another 15 min and washed by DPBST twice for 2 min.
5. Samples were blocked in blocking buffer at 4 °C overnight with gentle rotation.
6. Then samples were incubated with primary antibody solution at 4 °C overnight with gentle rotation.
7. Excessive primary antibody was removed by washing in (1) DPBST once for 5 min, (2) DPBST twice for 30 min, (3) DPBST once for 5 min with gentle rotation at room temperature.
8. Samples were incubated with secondary antibody solution at 4 °C overnight with gentle rotation.
9. Excessive secondary antibody was removed by washing in (1) DPBST once for 5 min, (2) DPBST twice for 30 min, (3) DPBST once for 5 min with gentle rotation at room temperature.

DAPI staining and confocal microscopy for combined FISH and IHC were performed same as FISH samples as indicated.

## Data Availability

All data generated or analyzed during this study are included in this published article.

## Abbreviations

FISH: Fluorescence in situ hybridization
HCR: Hybridization chain reaction
DPBS: Dulbecco’s phosphate buffered saline
IHC: Immunohistochemistry
FAA: Formaldehyde alcohol acetic acid
